# Unusual Stability of Messenger RNA in Snake Venom Reveals Gene Expression Dynamics of Venom Replenishment

**DOI:** 10.1371/journal.pone.0041888

**Published:** 2012-08-07

**Authors:** Rachel B. Currier, Juan J. Calvete, Libia Sanz, Robert A. Harrison, Paul D. Rowley, Simon C. Wagstaff

**Affiliations:** 1 Alistair Reid Venom Research Unit, School of Tropical Medicine, Liverpool, United Kingdom; 2 Laboratorio de Proteinomica Estructual, Instituto de Biomedicina de Valencia, Valencia, Spain; University of Crete, Greece

## Abstract

Venom is a critical evolutionary innovation enabling venomous snakes to become successful limbless predators; it is therefore vital that venomous snakes possess a highly efficient venom production and delivery system to maintain their predatory arsenal. Here, we exploit the unusual stability of messenger RNA in venom to conduct, for the first time, quantitative PCR to characterise the dynamics of gene expression of newly synthesised venom proteins following venom depletion. Quantitative PCR directly from venom enables real-time dynamic studies of gene expression in the same animals because it circumvents the conventional requirement to sacrifice snakes to extract mRNA from dissected venom glands. Using qPCR and proteomic analysis, we show that gene expression and protein re-synthesis triggered by venom expulsion peaks between days 3–7 of the cycle of venom replenishment, with different protein families expressed in parallel. We demonstrate that venom re-synthesis occurs very rapidly following depletion of venom stores, presumably to ensure venomous snakes retain their ability to efficiently predate and remain defended from predators. The stability of mRNA in venom is biologically fascinating, and could significantly empower venom research by expanding opportunities to produce transcriptomes from historical venom stocks and rare or endangered venomous species, for new therapeutic, diagnostic and evolutionary studies.

## Introduction

Snake venom is an evolutionary innovation contributing to the success of venomous snakes as proficient limbless predators. Venom consists of a complex mixture of proteins and peptides that have evolved from normal physiological proteins [1] into multi-isoform, multi-domain protein families with distinct biochemical targets. The collective spectrum of pharmacological specificities and biological potency of venom ensures rapid and efficient immobilisation, killing and digestion of a diverse range of prey species, irrespective of physiological differences [2]. The pathological consequences of snakebite also constitute an effective defence against predators and aggressors. An efficient venom production system is therefore important for venomous snakes to overcome their vulnerability following depletion of venom glands after a predatory or defensive snakebite.

Venom glands are modified parotid glands comprising a densely folded secretory epithelium consisting of several distinct cell types, including glandular secretory, mitochondria-rich, horizontal and ‘dark’ cells [3]–[5]. This abundance of secretory cells is required for rapid re-synthesis of venom proteins and other components to replenish venom stores after a bite. The systems for storage of venom following exocrine secretion depends upon the snake species, and include in (i) the central venom gland lumen, (ii) smaller tubular ductules, (iii) intracellular granules [3] and (iv) microvesicles within the lumen [6]. The dynamics of venom accumulation during synthesis is of great interest but little understood. Although early studies suggest total RNA and total protein levels peak at day 3 and days 4 to 8 days post venom expulsion respectively [7], little is known about the expression dynamics of individual venom components. This has been historically problematic because of the unpalatable need to sacrifice snakes (often rare, difficult to capture and CITES listed) to isolate mRNA from dissected venom glands. The observation of Chen *et al* [8], demonstrating that intact mRNA can be recovered, and toxins can be PCR-amplified and cloned from snake venoms and the venoms/skin secretions of other animals including Heloderma lizard [9], scorpion [10] and fire-bellied toads [11]–[13], has potential to resolve this research bottleneck. Exploiting these unusual observations, we have used venom as a resource and developed qPCR techniques as a tool to monitor the expression dynamics of mRNA encoding multiple venom toxin genes. Using the African Puff Adder (*Bitis arietans*) as a model viper species, we have characterised the expression profiles of venom genes in comparison with the protein composition and enzyme activity during venom synthesis. We also demonstrate that mRNA is a remarkably stable component of venom and discuss how this unusual phenomenon has potential to significantly empower venom research.

**Figure 1:**
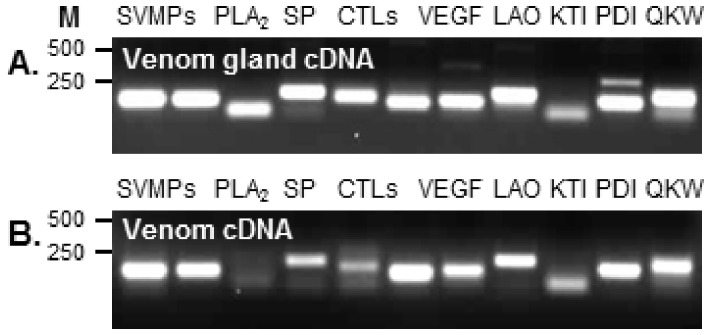
PCR amplification of cDNA constructed from venom gland and venom mRNA. Qualitatively similar PCR products were amplified from cDNA from venom gland (A) or venom (B), using primers complementary to *Bitis arietans* venom metalloproteinases (SVMP), phospholipase A_2_ (PLA_2_), serine protease (SP), C-type lectins (CTL), vascular endothelial growth factor (VEGF), L-amino acid oxidase (LAO), Kunitz inhibitors (KTI), protein disulphide isomerase (PDI) and QKW inhibitory peptides (QKW). Molecular weight markers (M) are shown to the left.

## Methods

### Venom Samples and Standards

All work performed on snakes in this study was conducted using protocols approved by the Liverpool School of Tropical Medicine Animal Welfare committee and performed under licence approved by the UK Home Office. Eight adult *Bitis arietans* specimens originating from Ghana (BaG) or Nigeria (BaN) were maintained in the herpetarium at the Liverpool School of Tropical Medicine under identical dietary and environmental conditions. Snakes were identified as BaG1, BaG2, BaG3, BaN1, BaN2, BaN3, BaN4 and BaN5. No venom was extracted from the animals for at least 25 days prior to the start of the study. The first venom extraction was referred to as mature venom. Thereafter, venom was extracted from the same individuals at four time points, referred to as day 0–1, day 0–3 and day 0–7, and immediately frozen at −20°C, lyophilised and stored at 4°C. Wet and dry masses of venom samples were recorded. Lyophilised venom samples were reconstituted in phosphate buffered saline (PBS) at their natural concentrations by re-suspension in volumes of PBS identical to that lost during lyophilisation. The venom yield for each extraction was calculated as the percentage of the highest quantity of venom yielded for each specimen across all venom extractions in this study. For gene expression analysis, PCR primer efficiency was tested by conventional PCR using standard samples including (i) venom gland cDNA from a *B. arietans* venom gland cDNA library constructed using methods described in Wagstaff *et al* [14] (as venom gland positive control) and (ii) cDNA synthesised from mRNA isolated from 10 mg of pooled mature venom (as venom positive control). Optimisation of quantitative PCR experiments was performed with mRNA isolated from 2 mg of pooled mature venom.

**Figure 2:**
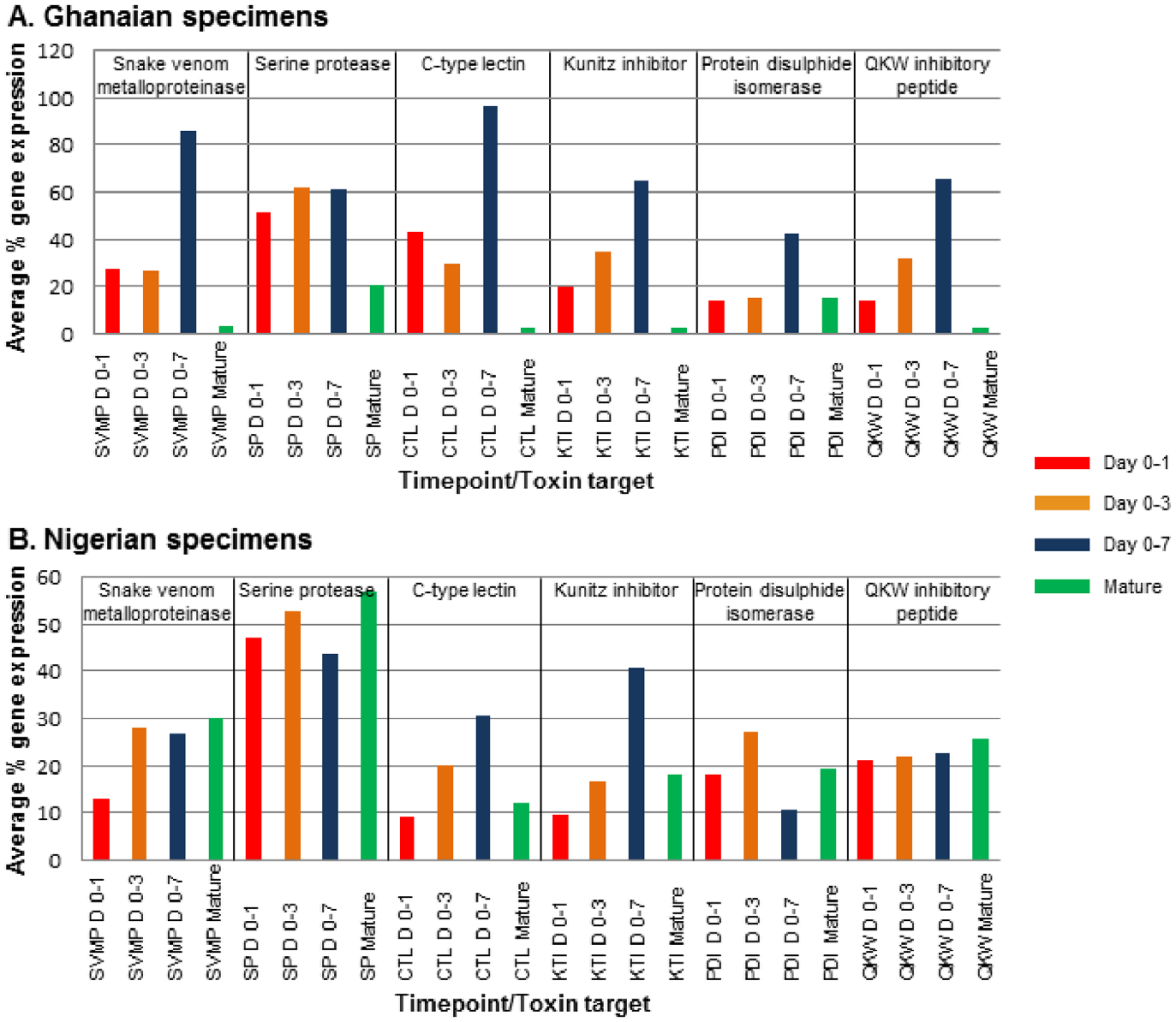
Venom mRNA expression profiles during venom re-synthesis. Relative expression profiles of six venom transcripts (snake venom metalloproteinase, serine protease, C-type lectin, Kunitz inhibitors, protein disulphide isomerase and QKW inhibitory peptide) across the time course of venom re-synthesis were determined by relative quantitative PCR (qPCR). The average fold changes in % venom protein gene expression for A) 3 Ghanaian *B. arietans* specimens and B) 5 Nigerian *B. arietans* specimens was normalised against three references genes (β actin, glyceraldehyde-3-phosphate dehydrogenase and heat shock protein) and indicate that the expression of venom protein genes peaks on day 0–3 to 0–7 (Red = day 0–1, orange = day 0–3, blue = day 0–7, green = mature venom).

### mRNA Extractions from Venom

Poly adenylated messenger RNA (mRNA) was purified from lyophilised venom using Dynabeads® mRNA DIRECT™ Kit (Dynal, Invitrogen) using the manufacturer’s protocol. Briefly, 2 mg of each lyophilised venom sample was reconstituted in 300 µl lysis/binding buffer (100 mM Tris-HCl pH 7.5, 500 mM LiCl, 10 mM EDTA pH 8, 1% LiDS and 5 mM dithiothreitol) and mixed with 50 µl magnetic oligo (dT)_25_ coated Dynabeads® at room temperature for 10 minutes. The mRNA-coated beads were magnetically separated from the unbound material, washed twice using 600 µl washing buffer A (10 mM Tris-HCl pH 7.5, 0.15 M LiCl, 1 mM EDTA and 1% LiDS) and once with 300 µl washing buffer B (10 mM Tris-HCl pH 7.5, 0.15 M LiCl, 1 mM EDTA). mRNA was eluted from beads in 10 µl of 10 mM Tris-HCl, pH 7.5 at 70°C for 2 minutes. The remaining unbound material was transferred back to fresh pre-washed magnetic beads and the mRNA isolation protocol was repeated to ensure complete capture of mRNA from venom. mRNA obtained from the first and second elution was pooled to obtain a total volume of 20 µl.

**Table 1:** Statistical analysis of relative gene expression by quantitative PCR.

Time point (I)	Time point (J)	Mean difference (I–J)	P-value
**Day 0–1**	Day 0–3	−0.149	1.000
	Day 0–7	−0.375	0.028*
	Mature	0.054	1.000
**Day 0–3**	Day 0–7	−0.226	0.515
	Mature	0.202	0.747
**Day 0–7**	Mature	0.429	0.008*

Fold changes in relative expression levels of venom genes of interest (raw data included in table S2). Analysis by regression analysis and Bonferroni post-hoc testing shows a statistically significant difference in gene expression between day 0–1 and 0–7, and day 0–7 and mature venom.

* indicates significant p-value (<0.05).

### cDNA Synthesis

cDNA was synthesised from 8 µl of eluted mRNA per reaction using Superscript® III first strand synthesis system (Invitrogen) according to the manufacturer’s instructions and stored at −20°C until required. To control for DNA contamination, reverse transcriptase negative controls were performed by substituting reverse transcriptase with 1 µl ultrapure water. Negative control reactions were included in conventional PCR to confirm the absence of amplicons arising from contaminating genomic DNA (data not shown).

**Figure 3:**
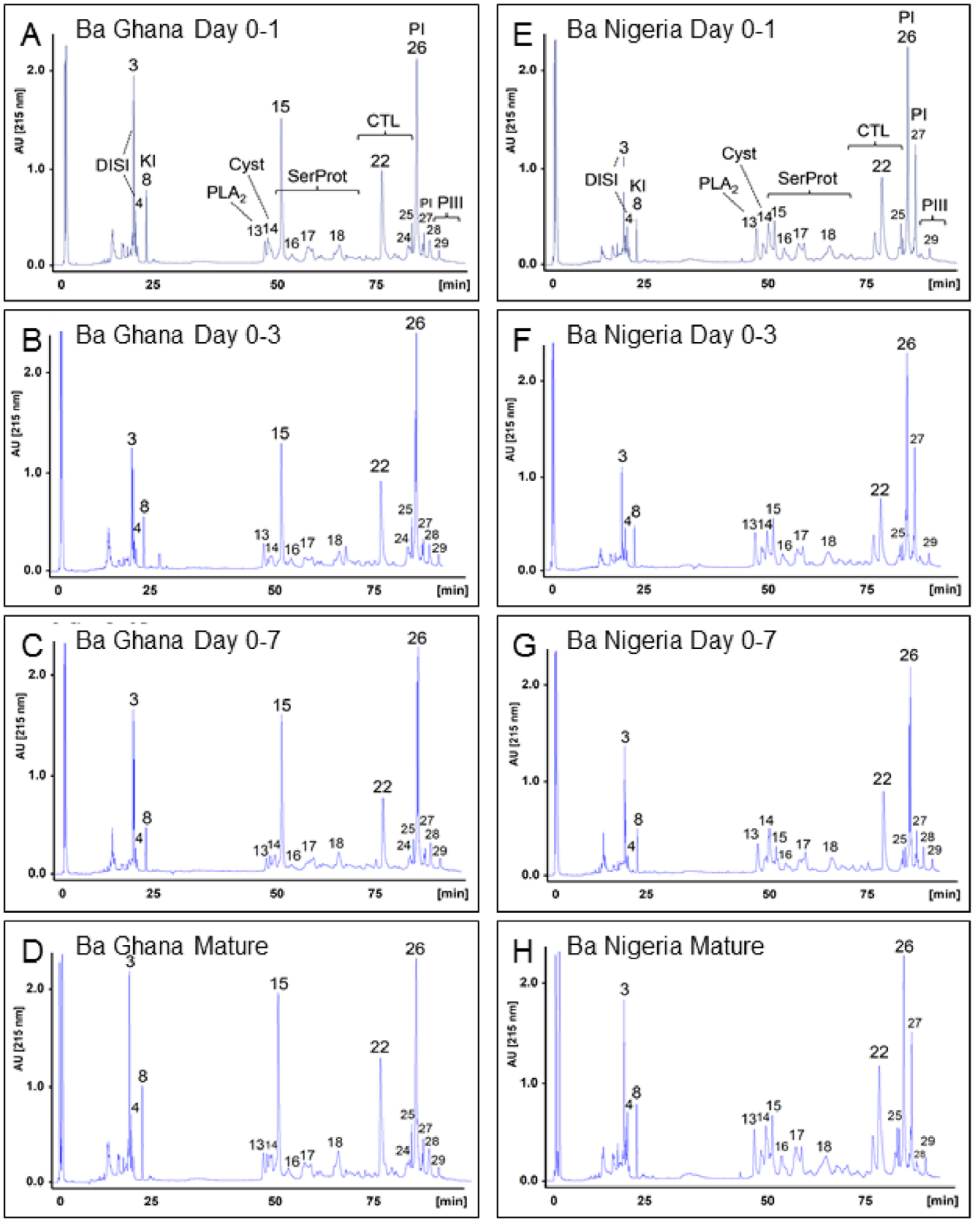
HPLC and mass spectrometry analysis of pooled venom samples from Ghana and Nigeria during venom re-synthesis. HPLC-MS/MS identification of proteins from pooled Ghanaian and Nigerian venom samples showed that very little quantitative changes in the protein composition of venom during protein re-synthesis. We identified by mass spectrometry a range of proteins including disintegrins (DISI, peaks 3, 4), Kunitz inhibitors (KI, peak 8), PLA_2_ (peak 13), cysteine-rich secretory proteins (Cyst, peak 14), serine proteases (SerProt, peaks 15–18), CTLs (peaks 22, 25) and PI (peak 26, 27) and PIII SVMPs (peak 29) which were present in all venom samples from day 0–1 to mature venom.

### Primer Design

Quantitative PCR primers were designed to amplify a range of venom protein-encoding gene targets with varied representation in our in-house *B. arietans* venom gland EST database including a class PII snake venom metalloproteinase, BAR00042, group II phospholipase A_2_, BAR00406, serine protease, BAR00034, C-type lectin, BAR00012, vascular endothelial growth factor, BAR00040, L-amino acid oxidase, BAR00017, Kunitz inhibitor, BAR00023, protein disulphide isomerase BAR00008 and QKW tri-peptide inhibitors of SVMPs, BAR00003 [15]. Three housekeeping genes were selected as reference transcripts: β-actin, glyceraldehyde 3-phosphate dehydrogenase (GAPDH) and heat shock protein – as used previously to improve accuracy of normalization [16]. The expression of reference genes was used as a baseline to which the expression of target genes was normalized. Reference gene primer pairs were designed on consensus sequences obtained from other snake species. Primer sequences are shown in table S1. Primer pairs were designed using Primer Select software (DNASTAR) complementary to sequences within the coding regions of specific genes to produce an amplicon of approximately 200 bp and with melting temperatures between 50 and 65°C. Primers were synthesised by Sigma Aldrich, UK.

**Figure 4:**
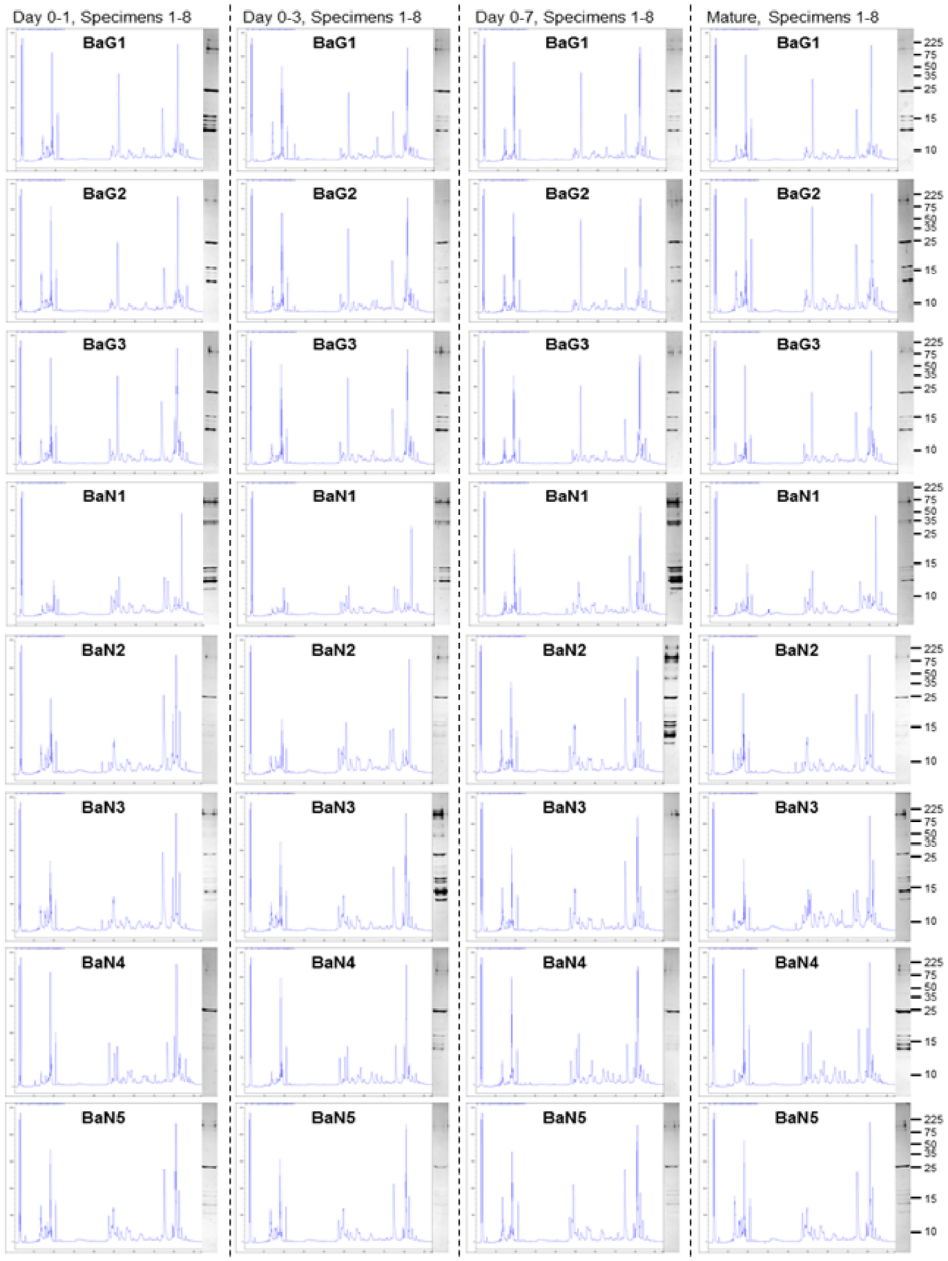
Individual venom protein profiles during venom re-synthesis by HPLC and 1D SDS-PAGE. Analysis of venom samples extracted on day 0–1, day 0–3, day 0–7 and mature venom for each individual specimen across the time course of venom re-synthesis by HPLC showed very little quantitative differences in protein profile. 1D-SDS-PAGE panels are shown to the right of HPLC profiles which confirm observations by HPLC. Molecular weight markers (M) are shown to the far right.

### Conventional PCR (PCR)

Conventional PCR was performed using the GoTaq® PCR Core system I (Promega). PCR reactions were prepared containing 2 µl venom cDNA, 10 µl 5×PCR buffer, 4 µl MgCl_2_, 1 µl dNTPs, 2.5 µl 10 mM 5′ primer, 2.5 µl 10 mM 3′ primer, 0.25 µl *Taq* polymerase and 27.75 µl PCR-grade water. As a venom gland positive control, 1 µl cDNA from a *B. arietans* venom gland cDNA library was used. Touch-down amplification was performed as follows: initial denaturation at 94°C for 5 min, 35 cycles of 94°C for 1 min, 66–55°C for 1 min, 72°C for 1 min with a final extension at 72°C for 7 min. Amplicons were visualised on a 1% TAE/agarose gel.

**Figure 5:**
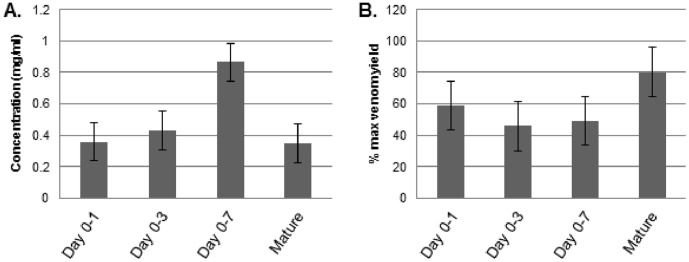
Correlation between natural venom protein concentration and venom yield. The natural protein concentration of venoms and venom yield of venoms extracted from day 0–1, 0–3, 0–7 were analysed. An inverse relationship between the protein concentration of venom (A) and the % maximum venom yield (B) over time was observed, indicating that venom protein concentration peaks between day 0–3 and day 0–7.

### Quantitative PCR (qPCR)

#### Optimisation of venom qPCR

Quantitative PCR experiments were conducted with reference to the Minimum Information for Publication of Quantitative Real-time PCR experiments (MIQE) guidelines [17] (table S1). Standard curves for each gene of interest and reference gene were performed to (i) obtain PCR reaction efficiencies and identify the optimal cDNA concentration required to obtain linear amplification. Melt curve analysis was used to assess the specificity of primers - a single post-amplification peak on the melt curve denoting a specific PCR product. To generate standard curves, 1 µg of cDNA synthesised from mRNA isolated from pooled mature venom was diluted across ten doubling dilutions. Reactions were prepared using the KAPA SYBR® FAST qPCR Kit (KAPA Biosystems, AnaChem) containing 1 µl venom cDNA template, 5.5 µl KAPA SYBR® FAST 2×qPCR master mix (including DNA polymerase, SYBR green fluorescent dye, MgCl_2_, dNTPs and stabilisers), 0.22 µl 10 mM 3′ primer, 0.22 µl 10 mM 5′ primer and 4.06 µl PCR-grade water. Amplifications were performed in duplicate alongside no template controls using a BioRad CFX 384 real-time PCR detection system with an initial denaturation step of 95°C for 3 minutes followed by 40 cycles of 95°C for 10 seconds, 55°C for 30 seconds. Melt curve analysis was performed by heating the amplicon at 95°C for 10 seconds followed by repeated cycles of denaturation from 55°C to 95°C at 0.5°C increments. Examples of the standard and melt curve analysis for snake venom metalloproteinase and C-type lectin are shown in figure S1. The most efficient and specific primer pairs were selected for qPCR.

**Figure 6:**
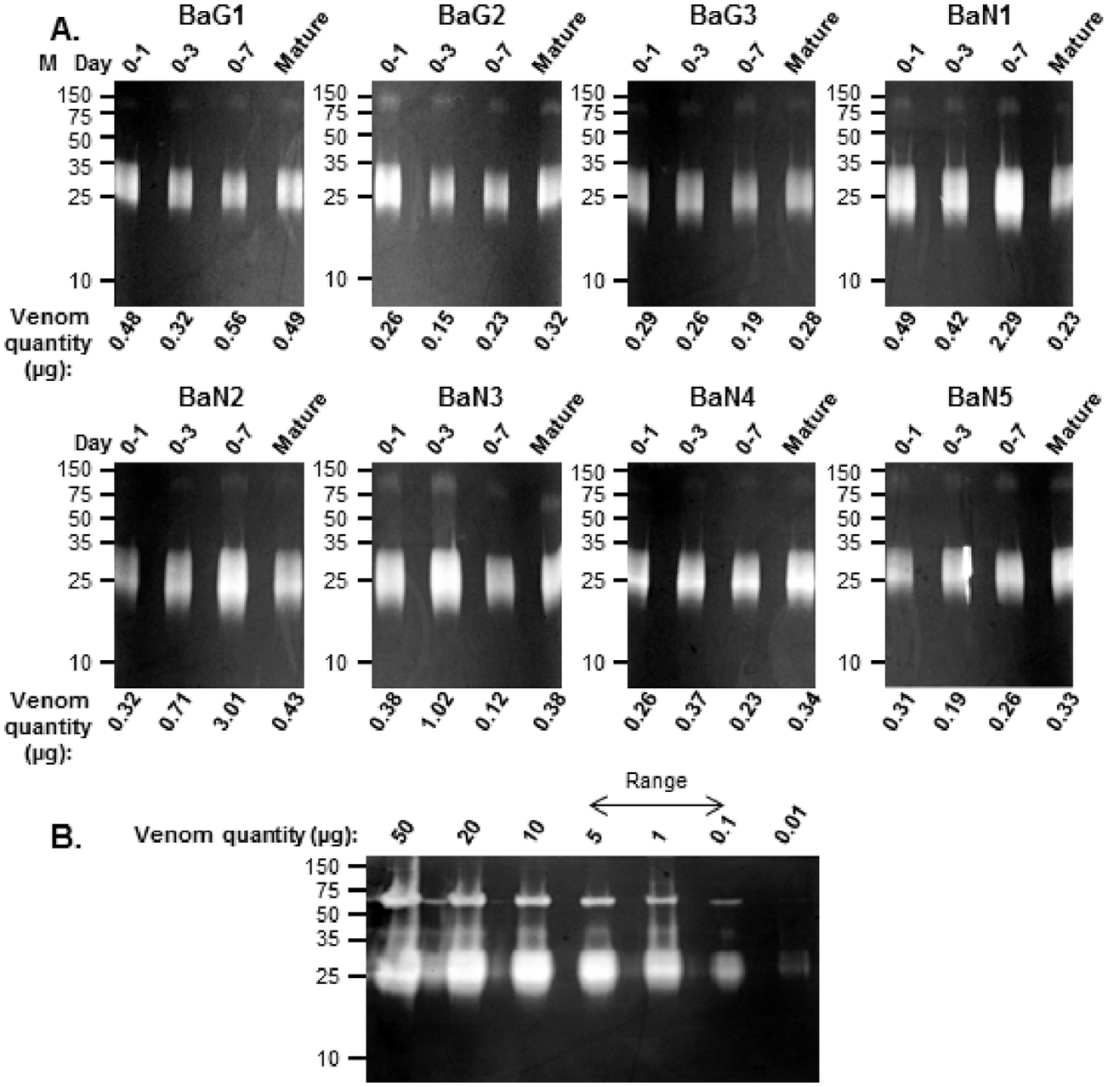
Venom enzyme activity profiles during venom re-synthesis. Gelatin zymography of venom samples extracted at day 0–1, 0–3, 0–7 and mature venom and reconstituted at natural protein concentration was used to assess enzyme activity of venom (A). The enzymatic degradation of substrate by venom extracted on day 0–1 was equal to mature venom. Panel B shows a range-finding zymogram which shows that the dynamic range of this assay (arrow above indicate the range of venom quantity used in panel A). Molecular weight markers (M) are shown to the left.

#### qPCR amplification of unknown venom samples and analysis

11 µl KAPA SYBR® FAST qPCR reactions (KAPA Biosystems, AnaChem) containing 1 µg of each cDNA sample were prepared in triplicate alongside no template controls as previously described in the standard curve protocol. Amplification cycles were analysed with the BioRad CFX manager software (version 1.5) in gene expression mode. Fold expression change for each gene of interest was calculated using the ΔΔCt comparative method [16], [18] normalised against three reference genes: β-actin, GAPDH and heat shock protein. An example of the raw data showing relative fold changes in gene expression is provided in figure S2 and table S2. The data were transformed onto a log scale and analysed by univariate analysis of variance using PASW statistics version 18 (SPSS Inc.) [19] fitting individual snake, time point and venom protein as fixed effects. Differences in venom production attributable to time points were investigated allowing a Bonferroni correction for multiple comparisons. Following statistical analysis, raw data for each individual specimen was standardised by converting fold expression to a percentage of the maximum expression value for each specimen. At each time point, the expression of each toxin target was averaged for all 8 specimens in the study.

**Figure 7:**
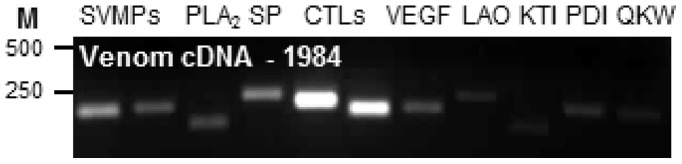
Prolonged stability of mRNA in lyophilised venom. PCR amplification of a range of venom protein transcripts including snake venom metalloproteinases (SVMP), phospholipase A_2_ (PLA_2_), serine protease (SP), C-type lectins (CTL), vascular endothelial growth factor (VEGF), L-amino acid oxidase (LAO), Kunitz inhibitors (KTI), protein disulphide isomerase (PDI) and QKW inhibitory peptides (QKW) from mRNA isolated from a venom sample extracted and lyophilised in 1984.

### Reverse-phase HPLC Separation of Proteins and Mass Spectrometry Protein Identification

1 mg of each venom sample was dissolved in 100 µl 0.05% trifluoroacetic acid (TFA) and 5% acetonitronitrile. Insoluble material was removed by centrifugation at 13,000 g for 10 minutes at room temperature. Soluble venom proteins were separated by reverse-phase HPLC using a Teknokroma Europa C18 (0.4 cm×25 cm, 5 mm particle size, 300 Å pore size) column and an Agilent LC 1100 High Pressure Gradient System equipped with DAD detector and micro-autosampler. The flow-rate was set to 1 ml/min and the column was developed with a linear gradient of 0.1% TFA in water (solution A) and acetonitrile (solution B), isocratically (5% B) for 5 min, followed by 5–25% B for 20 min, 25–45% B for 60 min, and 45–70% for 10 min. Protein detection was carried out at 215 nm with a reference wavelength of 400 nm and peaks were collected manually. Proteins were analysed by SDS-PAGE and the protein bands identified by in-gel tryptic digestion, MALDI-TOF peptide mass fingerprinting (using an Applied Biosystem’s Voyager DE-Pro instrument) against the SwissProt/TrEMBL database plus previously assigned peptide ion sequences from *Bitis* species [20]–[21]. *De novo* peptide ion sequencing was performed by electrospray ionization (ESI) tandem mass spectrometry using an Applied Biosystems QTrap™ 2000 mass spectrometer [22]. Amino acid sequence similarity searches were performed against the available databanks using the BLAST program [23].

### One-dimensional SDS-PAGE and Gelatin Zymography

Venom samples reconstituted to their natural concentrations were prepared in reducing SDS-PAGE sample buffer, separated on 1 mm 15% SDS-PAGE gels according to the manufacturer’s recommendations (BioRad) and silver stained using a rapid staining protocol [24]. For zymography, venoms prepared in non-reducing SDS-PAGE buffer were separated on 0.75 mm polyacrylamide gels (2.5 ml distilled water, 2.5 ml 1.5 M Tris pH 8.8, 2.5 ml 40% Bis-Acrylamide, 50 µl 10% SDS, 150 µl 10% ammonium persulphate and 7.5 µl TEMED) co-polymerised with 2.5 ml 10 mg/ml molten gelatin (Sigma Aldrich, UK). Following electrophoresis, gels were washed with renaturing buffer (2.5% Triton X-100) for 30 minutes at room temperature with agitation to remove SDS. Gels were then washed with distilled water and incubated overnight at 37°C with gentle agitation in 50 ml developing buffer/gel (1 M Tris pH.8, 1 M CaCl_2_) to activate metal ion-dependent venom proteases. Gels were stained with Coomassie Blue R-250 and destained until clear areas, indicating enzyme activity, were observed.

## Results

### Quantity and Quality of mRNA Recovered from Venom

An average yield of 46.2 ng (±13 ng std. dev) mRNA was purified from the 2 mg venom samples (approximately 7% of the typical venom yield) and, separately, we recovered 420 ng from 10 mg lyophilised venom. These venom mRNA recovery figures varied little from days 0–1 to mature venom. The average 260/280 absorbance ratios of the venom mRNA samples was 2.43±0.57. These results demonstrate that potentially large quantities of mRNA, of high quality, can be reproducibly recovered from *B. arietans* venom. More than adequate amounts of mRNA were recovered from each venom sample for downstream qPCR analysis: a representative cDNA synthesis reaction yielded 22.74 µg (±3.03 µg std. dev) of cDNA (20 µl) per reaction from 18.5 ng of mRNA (8 µl) - amounts easily sufficient for 20 qPCR reactions.

### Amplification of Similar Products from PCR of cDNA Originating from Venom or Venom Gland mRNA

We designed PCR primers complementary to representatives of the spectrum of proteins expressed in viper venom such as the highly toxic snake venom metalloproteinases and serine proteases and proteins function such as protein disulphide isomerase and QKW inhibitory peptides. The PCR products obtained by conventional PCR with *B. arietans* venom gland cDNA (Figure. 1A) were qualitatively similar to those amplified from venom cDNA, but quantitative differences in the intensity of amplicons were observed (Figure. 1B). These observations should be interpreted in context of cDNA samples; venom gland cDNA was extracted from venom glandular tissue pooled from 10 specimens sacrificed 3 days after venom extraction when the transcriptional activity of the venom gland is thought to peak. In contrast, the venom cDNA was prepared from a pool of venom collected from 15+ snakes that had been ‘milked’ on numerous occasions - which could account for minor quantitative differences in amplification observed. The spectrum of mRNA amplified from venom appeared to be broadly similar to venom gland – strongly indicating that mRNA from venom is representative of the toxin and non-toxin composition of mRNA from venom gland tissue, irrespective of their high or low representation in the transcriptome.

### Venom Protein Expression Profiles during Venom Re-synthesis

Having confirmed venom as a valid biological resource of most protein-encoding mRNA tested in this study, we collected venom samples from eight puff adders (of varying size/age, sex and geographic origin) at intervals after venom the initial extraction of mature venom (day 0–1, 0–3 and 0–7) to investigate the time scale of venom re-synthesis. We identified a peak between day 0–3 and 0–7 in the mRNA expression of snake venom metalloproteinase, serine protease, C-type lectin, Kunitz inhibitor, protein disulphide isomerase and QKW inhibitory peptides (Figure. 2). Statistical analysis of relative gene expression data revealed highly significant differences in expression between individual snakes (p = 0.001). Previous studies illustrated that venom from *B. arietans* varies considerably in protein composition, enzyme activity and immunoreactivity between specimens of the same and different geographical origins, possibly in response to environmental pressures [25]. Our findings here suggest that venoms from individual specimens also vary in terms of (i) toxin expression levels and (ii) expression of toxins at different time points after venom expulsion (p = 0.006). Individual toxin-expression expression profiles over time are shown in Table 1 with p values Bonferroni-corrected for multiple comparisons, demonstrating that the relatively lower mRNA expression levels observed in day 0–1 and mature venom samples were statistically significant. The standard error in statistical analysis was 0.131 for all samples.

### Protein Profiles and Venom Activity during Venom Re-synthesis

The same *B. arietans* venoms samples used in relative gene expression analyses were fractionated by HPLC to examine the protein composition of venom during the course of venom re-synthesis (Figure. 3). HPLC protein profiles of pooled venoms extracted at different time points from Ghanaian specimens (Figure 3 A–D) and Nigerian specimens (Figure 3 E–H) revealed very little quantitative differences in venom composition and relative proportions of venom proteins within the time-course of venom replenishment examined (day 0–1 to maturity). We further observed very little quantitative changes in venom protein composition between individuals (Figure 4).

Although we observed little quantitative differences in the protein composition of venom, changes in the natural concentration of venom were apparent. Overall, the natural concentration of venom proteins was highest in venoms extracted from day 0–3 to 0–7 (Figure. 5A) compared to any other time of the venom re-synthesis cycle. This peak in protein concentration on day 0–3 to 0–7 peak (Figure. 5A) inversely correlated with venom yield (Figure. 5B), indicating that the larger volumes of venom delivered by *B. arietans* later in the venom production cycle do not reflect a temporally-exponential increase in toxin expression but the greater production of saliva, mucus or other secretions. Also, and consistent with the gene-expression data, the results suggest that the majority of venom proteins appear to be synthesised rapidly following extraction. This result also correlates with gelatin zymography assays, used to assess venom proteolytic activity during the time course of venom re-synthesis. Our results showed that the enzyme activity of venom did not appear to change over the time course of venom re-synthesis and, most notably, venom extracted on day 0–1 was equally efficient at degrading gelatin substrate as mature venom (Figure. 6).

## Discussion

Previous work by Chen *et al* identified mRNA as a frequent and stable component of venoms [8]. Here we demonstrate that this discovery offers an alternative approach to investigate venom transcriptomics and gene expression which circumvents the conventional need to sacrifice animals for venom gland tissue. We utilised venom as a source of mRNA, in combination with validated quantitative PCR protocols to monitor transcription of venom toxin and non-toxin genes in real time in response to their requirement for venom re-synthesis. We also show that the protein-coding spectrum of mRNA is the same for venom and venom gland, which strongly suggests that the venom transcriptome is an accurate representative of the venom gland transcriptome.

The prolonged presence/stability of mRNA in snake venom is very unusual as in most organisms, mRNAs are typically highly labile with rapid turnover rates [26], [27]. This natural instability of mRNA is biologically important as it permits the cell to adapt and respond to changing environmental or developmental cues requiring rapid up or down-regulation of gene expression [28]. Our demonstration that mRNA can be detected in venom at each time point during the complete time course of venom synthesis is remarkable because we would expect snake venom glands to present a highly unfavourable environment for mRNA preservation due to the diverse array of destructive nucleases and phosphodiesterases [29], and naturally acidic conditions [5]. In an extreme extension of this investigation, we also report that mRNA encoding snake venom metalloproteinase, serine protease, C-type lectin, Kunitz inhibitor, protein disulphide isomerase and QKW inhibitory peptide was PCR amplified from *B. arietans* venom which was extracted and lyophilised in 1984 (Figure. 7).

Understanding the stability and potential role of mRNA in venoms is the focus of future work. We expect that multiple factors are involved in maintaining mRNA stability. Firstly, lyophilisation and preservation of venom may initially prevent degradation of venom components, although mRNAs found specifically in venom could have unique properties leading to an unusual long-term stability. Physiochemical properties of venom, including the presence of high concentrations of citrate [30]–[32] and a weakly acidic pH [33]–[34] [5], may provide universal stability to many venom components. Finally, there is evidence for toxin-specific inhibitors in venom such as the QKW tri-peptide inhibitors of SVMPs [15], and such specific or non-specific inhibitors may play a role in stabilising other components of venom, including mRNAs. Overall, the venom microenvironment appears to impart unusual stability upon mRNA; observations that can be exploited to significantly expand opportunities to research venoms and venom biology.

In the first study of its kind, we applied this technique to investigate the time scale of venom synthesis and established that expression of several venom transcripts peaks between days 3 to 7 of the cycle of protein replenishment. Analysis of venom transcript and protein expression profiles suggests that there is a close temporal correlation between transcription and incorporation of proteins into venom during venom synthesis. Our results also demonstrate that biologically active venom proteins, such as the tissue-destructive snake venom metalloproteinases, are present in venom within one day of venom depletion. The speed at which venom is replenished reflects the critical requirement for snakes, as limbless predators, to rapidly re-synthesise functional venom for both predatory and defensive purposes.

The qPCR transcription profiling indicates that re-synthesis of venom components examined in this study occurs in parallel rather than by a coordinated serial expression of different venom protein families. This appears to be contrary to early immunohistochemical reporting the asynchronous regeneration of distinct venom protein families [35]–[37], that utilised very different methods from those used here. Since the venom proteins we surveyed exhibit a wide range of pharmacological/physiological functions, our observations suggest that the dynamics of venom replenishment may not be dependent on the biological roles of venom proteins.

Although the evolution of venom protein-coding sequences has been extensively studied, we currently have very little understanding of the machinery involved in coordinating venom expression and gene regulation. This is important for our understanding of both the biology of venomous animals and the evolution of toxicity. Snake venom has evolved into a highly complex mixture of proteins and peptides by mechanisms of gene duplication and selection from ‘normal’ ancestral non-toxin homologues, and is continually subjected to adaptive evolutionary pressures involving gene recruitment and domain loss events [38], [39]. Understanding how newly recruited toxin prototypes are placed under the control of the venom production apparatus is the focus of future work aimed at characterising the specific regulatory machinery responsible for robust, yet selective expression of toxins in venom.

## Supporting Information

Figure S1 Optimisation of venom quantitative PCR. Representative standard curves for snake venom metalloproteinase (SVMP) and C-type lectin (CTL) (1Ai and Aii) show high efficiency amplification of 94.0 and 96.6% respectively. Representative melt curves for SVMP and CTL amplicons showing a single melt peak indicating a single specific amplicon (1Bi and Bii).
(TIF)

Figure S2 Raw data following gene expression analysis by quantitative PCR. Gene expression analysis conducted using the BioRad CFX manager software. Relative gene expression was calculated from the cycle time (Ct value) using the ΔΔCt method. Expression profiles for all individual specimens in the study are shown illustrating fold changes in the expression of six genes of interest from day 0–1 to mature venom, normalised to three reference genes; β actin, glyceraldehyde-3-phosphate dehydrogenase and heat shock protein (Red = day 0–1, orange = day 0–3, blue = day 0–7, green = mature venom).
(TIF)

Table S1 Minimum information for Publication of Quantitative Real-time PCR Experiments (MIQE) guidelines. Guidelines published by Bustin *et al* 2009 were referred to in order to ensure accuracy and reliability of quantitative PCR data.
(DOCX)

Table S2 Raw individual relative gene expression data generated by quantitative PCR. Raw qPCR data generated from relative expression analysis to show fold changes in expression of venom genes of interest, including snake venom metalloproteinase (SVMP), serine protease (SP), C-type lectin (CTL), Kunitz inhibitors (KTI), protein disulphide isomerase (PDI) and QKW inhibitory peptides (QKW).
(DOCX)
