# Supplementary figures and images for: Unusual Stability of Messenger RNA in Snake Venom Reveals Gene Expression Dynamics of Venom Replenishment
Source: PLoS One. 2012 Aug 7;7(8):e41888. doi: 10.1371/journal.pone.0041888 (PMC3413681; doi:10.1371/journal.pone.0041888)

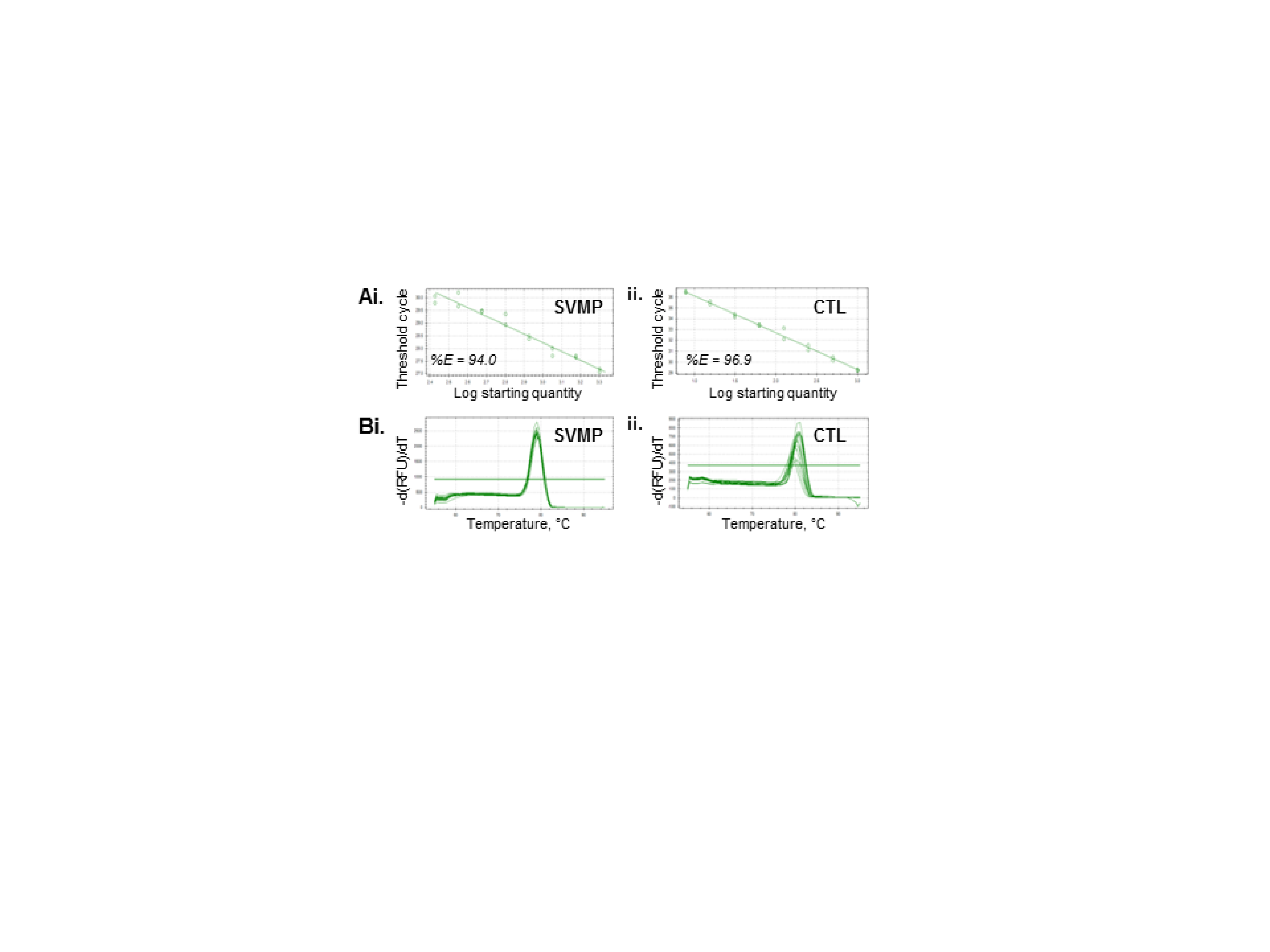

Supplement: Figure S1 — Optimisation of venom quantitative PCR. Representative standard curves for snake venom metalloproteinase (SVMP) and C-type lectin (CTL) (1Ai and Aii) show high efficiency amplification of 94.0 and 96.6% respectively. Representative melt curves for SVMP and CTL amplicons showing a single melt peak indicating a single specific amplicon (1Bi and Bii). (TIF) [file pone.0041888.s001.tif]

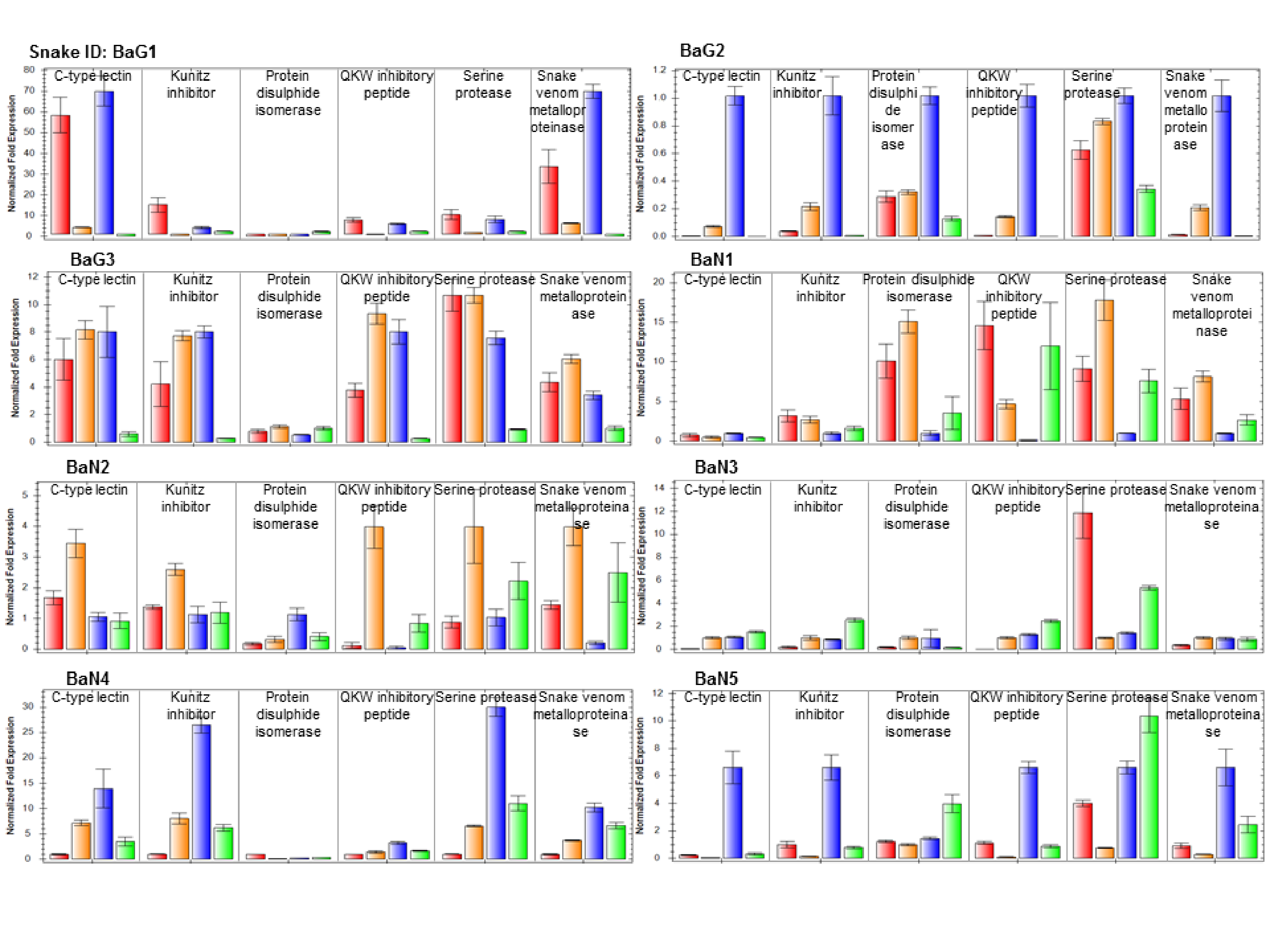

Supplement: Figure S2 — Raw data following gene expression analysis by quantitative PCR. Gene expression analysis conducted using the BioRad CFX manager software. Relative gene expression was calculated from the cycle time (Ct value) using the ΔΔCt method. Expression profiles for all individual specimens in the study are shown illustrating fold changes in the expression of six genes of interest from day 0–1 to mature venom, normalised to three reference genes; β actin, glyceraldehyde-3-phosphate dehydrogenase and heat shock protein (Red = day 0–1, orange = day 0–3, blue = day 0–7, green = mature venom). (TIF) [file pone.0041888.s002.tif]
