# Supplementary material for: Unusual Stability of Messenger RNA in Snake Venom Reveals Gene Expression Dynamics of Venom Replenishment
Source: PLoS One. 2012 Aug 7;7(8):e41888. doi: 10.1371/journal.pone.0041888 (PMC3413681; doi:10.1371/journal.pone.0041888)
Supplement: Table S1 — Minimum information for Publication of Quantitative Real-time PCR Experiments (MIQE) guidelines. Guidelines published by Bustin et al 2009 were referred to in order to ensure accuracy and reliability of quantitative PCR data. (DOCX) [file pone.0041888.s003.docx]

| **Supplementary table 1: Minimum information for Publication of Quantitative Real-time PCR Experiments (MIQE)** | | | | |
| --- | --- | --- | --- | --- |
| **Experimental design:** | | | | |
| *Number within each group* | 8 individual *Bitis arietans* specimens (originating from Ghana or Nigeria) were used in the study. | | | |
| *Location where assay carried out* | Laboratories of the Alistair Reid Venom Unit of the Molecular and Biochemical Parasitology Research Group, Liverpool School of Tropical Medicine. | | | |
| *Acknowledgement of authors’ contributions* | All experiments were carried out by RB Currier. | | | |
| **Sample:** | | | | |
| *Description* | Lyophilised venom extracted from 8 individual adult *Bitis arietans* specimens originating from Ghana or Nigeria (historical snake ID numbers: BaG2, BaG4, BaLZS1, BaN11, BaN60, BaN68, BaN80 and BaN81). | | | |
| *Volume/mass sample of processed* | 2-10mg lyophilised venom. | | | |
| *Processing procedure* | Once extracted, venom was frozen at -20°C before being lyophilised for 3-4 hours. | | | |
| *If frozen, how and how quickly?* | Fresh venom samples were frozen immediately after extraction. | | | |
| *Sample storage conditions and duration* | Freeze-dried venom samples were stored at 4°C. | | | |
| **Nucleic acid extraction:** | | | | |
| *Procedure and/or instrumentation* | mRNA extraction | | | |
| *Name of kit* | Dynabeads® mRNA DIRECT™ kit (Dynal, Invitrogen, UK). | | | |
| *Details of DNase or RNase treatment* | DNase treatment was performed on a standard sample (pooled mature venom). Conventional PCR was carried out and products were run on a 1% agarose gel. There were no differences in PCR products between DNase and non DNase treated mRNA samples. | | | |
| *Contamination assessment* | Reverse transcriptase negative controls for all toxin, non-toxin and reference genes were carried out using a standard mRNA sample (isolated from pooled mature venom). Amplicons from conventional PCR were run on a 1% agarose gel. No products were visible indicating that there was no contamination. | | | |
| *Nucleic acid quantification* | The concentration of mRNA extracted from venom was measured using the Nanodrop spectrophotometer. | | | |
| *Instrument and method* | Nanodrop spectrophotometer, Thermo Scientific. | | | |
| *Purity(A_260_/A_280_)* | OD_260/280_ of a control sample (pooled mature venom mRNA) measured using a Nanodrop. The OD_260/280_ reading of mRNA extracted from 10mg lyophilised *B. arietans* venom was 4.67. | | | |
| *Inhibition testing* | Standard curves of cDNA dilutions (including cDNA concentrations of up to 2µg/µl) were sufficient to detect the presence of PCR inhibitors introduced in nucleic acid preparation. | | | |
| **Reverse transcription:** | | | | |
| *Complete reaction conditions* | 1µl Primer (50µM oligo dT) and 1µl 10mM dNTP mix were added to each mRNA sample. The cDNA synthesis master mix contained (per reaction): 2µl 10x RT buffer, 4µl 25mM MgCl_2_, 2µl 0.1M DTT, 1µl RNaseOUT (40U/µl), 1µl Superscript III RT (200U/µl). 1µl RNase H was added to each reaction following termination of cDNA synthesis to degrade any RNA template. | | | |
| *Amount of RNA and reaction volume* | 8µl 2-5ng/µl mRNA was added to each 20µl reaction volume. | | | |
| *Reverse transcriptase and concentration* | Superscript® III reverse transcriptase (Invitrogen) at a concentration of 200U/µl. | | | |
| *Temperature and time* | First, samples were incubated for 5 minutes at 65°C and placed on ice for 1 minute. Following addition of the mastermix, samples were incubated for 50 minutes at 50°C. The reactions were terminated following incubation at 85°C for 5 minutes. Following addition of RNase H, samples were incubated at 37°C for 20 minutes. | | | |
| *Manufacturer of reagents and cat. numbers* | Superscript® III first strand synthesis system manufactured by Invitrogen (cat. number 18080-051). | | | |
| *Storage conditions of cDNA* | cDNA was stored at -20°C. | | | |
| **qPCR target information:** | | | | |
| *Amplicon length* | Amplicon lengths for toxin, non-toxin and reference gene primers:  Snake venom metalloproteinase – 150  Serine protease – 195  C-type lectin – 131  Kunitz inhibitor – 80  Protein disulphide isomerase – 160  QKW inhibitors – 181  β-actin – 156  GAPDH – 158  Heat shock protein - 148 | | | |
| *Secondary structure analysis* | Secondary structures were avoided during primer design. | | | |
| **qPCR oligonucleotides:** | | | | |
| *Primer sequences* | **Cluster number** | **Protein group** | **Forward primer sequence** | **Reverse primer sequence** |
|  | BAR00042 | SVMP | ATACTGCGTGGTCTAGAAATGTGG | AGCGCCTGTGAATAACTGAGC |
|  | BAR00034 | SP | AGGAGGCGAGGAGAAGAGACG | TTCCGCCCCATCCCATAATAC |
|  | BAR00012 | CTL | GCTCCGGCTTGCTGGTCGTGTTC | TCGATCGGCCCAGGTCTTCTCTAC |
|  | BAR00023 | KTI | GGGCCTATATCCGTTCCTTCTT | ATTCCCATAGCATCCACCATAAAA |
|  | BAR00008 | PDI | CCCGAATATTCTGGTGGAGT | AAATTGTTGGGCGAGTTCTG |
|  | BAR00003 | QKW | TGCGCCCCCAAATCCTCCTA | TGGCATACGCAGCTGGTTTACTCA |
|  | - | β-actin | CTCAGAGTCGCCCCGGAAGAACAT | AGAGGCGTACAGGGAGAGCACAGC |
|  | - | GAPDH | GAATATCATCCCAGCATCCACAGG | CATCATACTTCGCCGGTTTCTCTA |
|  | - | Heat shock protein | CTGCCAGAAGATGAAGATGAAAAG | CAATACAACACGGGGAAGAGACTA |
| *Manufacturer of oligonucleotides* | Sigma, UK. | | | |
| *Purification method* | Desalting | | | |
| **qPCR protocol:** | | | | |
| *Complete reaction conditions* | 1µl cDNA sample per reaction was added to 4µl ultrapure water. 6µl KAPA SYBR® FAST qPCR master mix was added to the diluted cDNA sample. | | | |
| *Reaction volume and amount of cDNA* | 11µl reaction volume containing 1µl cDNA. | | | |
| *Primer, Mg^2+^ and dNTP concentrations* | Primers were added to the master mix from 10µM solutions to produce a final concentration of 200nM in the master mix. There was no deviation in Mg^2+^ and dNTP to those provided within the manufacturer’s mastermix. | | | |
| *Polymerase identity and concentration* | There was no deviation in the concentration of KAPA SYBR® DNA polymerase to that provided within the manufacturer’s mastermix. | | | |
| *Buffer/kit identity and manufacturer* | KAPA SYBR® FAST qPCR kit, KAPA Biosystems, AnaChem. | | | |
| *Additives (SYBR green I, DMSO etc)* | SYBR green I was contained within the manufacturer’s mastermix. | | | |
| *Manufacturer of plates and cat. number* | BioRad, UK (Cat. HSP-3805) | | | |
| *Complete thermocycling parameters* | The following thermocycling protocol was used: enzyme activation 95°C, 3 minutes, [denaturation 95°C, 10 seconds, anneal/extend 55°C, 30 seconds] x 40 cycles. Melt curve were performed following amplification: 10 sec (95°C), ramping from 55°C to 95°C at 0.5°C increments. | | | |
| *Reaction setup* | Manual. | | | |
| *Manufacturer of qPCR instrument* | BioRad CFX 384 real time PCR detection system manufactured by BioRad. | | | |
| **qPCR validation:** | | | | |
| *Evidence of optimisation* | PCR with a temperature gradient from 50-65 ºC using all primer pairs was performed in order to determine optimal annealing temperature. An average optimal temperature for all primer pairs was calculated to be 55ºC. | | | |
| *Specificity (gel, sequence, melt or digest)* | Melt curves for example primer pairs are provided in supplementary figure 1. | | | |
| *For SYBR green I, C_q_ of the NTC* | All C_q_s for NTCs were negative indicating no DNA contamination. | | | |
| *Calibration curves with slope and y intercept* | Standard curves example primer pairs are provided in supplementary figure 1. | | | |
| *PCR efficiency calculated from slope* | Amplification efficiencies (%) -  Snake venom metalloproteinase: 94.0  Serine protease: 28.0  C-type lectin: 96.9  Kunitz inhibitor: 100.7  Protein disulphide isomerase: 102.0  QKW inhibitory peptide: 91.2  β-actin: 62.2  GAPDH: 51.7  Heat shock protein:89.5 | | | |
| **Data analysis:** | | | | |
| *qPCR analysis program (source, version)* | BioRad CFX manager version 1.5. | | | |
| *Method of C_q_ determination* | Single threshold. | | | |
| *Number/choice of reference genes* | 3; β-actin, GAPDH and heat shock protein were used as reference genes. | | | |
| *Description of normalised method* | The ΔΔC(t)) method for normalized relative gene expression was used. | | | |
| *Number of biological replicates* | 8 individual West African *B. arietans* specimens were used in this study. | | | |
| *Repeatability (intra-assay variation)* | To assess intra-assay repeatability, samples used for standard curve analysis were run in duplicate. All unknown samples were run in triplicate. | | | |
| *Reproducibility (inter-assay variation, CV)* | The venom extraction time course was repeated for all available specimens used in the study. mRNA extraction, cDNA synthesis and qPCR were performed under identical conditions as in the first time course. Relative expression analysis from both runs was in agreement. | | | |
| *Statistical methods for results significance* | Multiple regression analysis and Bonferroni post-hoc testing. | | | |
| *Software (source, version)* | PASW Statistics, SPSS Inc. Version18. | | | |
