# Supplementary material for: Unusual Stability of Messenger RNA in Snake Venom Reveals Gene Expression Dynamics of Venom Replenishment
Source: PLoS One. 2012 Aug 7;7(8):e41888. doi: 10.1371/journal.pone.0041888 (PMC3413681; doi:10.1371/journal.pone.0041888)
Supplement: Table S2 — Raw individual relative gene expression data generated by quantitative PCR. Raw qPCR data generated from relative expression analysis to show fold changes in expression of venom genes of interest, including snake venom metalloproteinase (SVMP), serine protease (SP), C-type lectin (CTL), Kunitz inhibitors (KTI), protein disulphide isomerase (PDI) and QKW inhibitory peptides (QKW). (DOCX) [file pone.0041888.s004.docx]

|  |  | **Snake ID** | | | | | | | |
| --- | --- | --- | --- | --- | --- | --- | --- | --- | --- |
| **Toxin** | **Time point** | **BaG1** | **BaG2** | **BaG3** | **BaN1** | **BaN2** | **BaN3** | **BaN4** | **BaN5** |
|  | Day 0-1 | 33.60321 | 0.01339 | 4.35748 | 5.36035 | 0.24207 | 0.37842 | 1.00000 | 0.92666 |
| **SVMP** | Day 0-3 | 6.30138 | 0.20878 | 6.04173 | 8.15441 | 1.04417 | 1.00000 | 3.71470 | 0.29722 |
|  | Day 0-7 | 69.91616 | 1.01773 | 3.41959 | 1.00000 | 0.10252 | 0.95119 | 10.24636 | 6.61914 |
|  | Mature | 0.14966 | 0.00485 | 1.00000 | 2.64833 | 1.66413 | 0.89011 | 6.62833 | 2.45152 |
|  | Day 0-1 | 10.48848 | 0.62434 | 10.67630 | 9.11500 | 0.34854 | 11.84324 | 1.00000 | 4.01206 |
| **SP** | Day 0-3 | 1.88829 | 0.83287 | 10.68738 | 17.73604 | 2.08213 | 1.00000 | 6.57814 | 0.75083 |
|  | Day 0-7 | 8.28376 | 1.01773 | 7.57303 | 1.00000 | 0.46168 | 1.43584 | 30.00774 | 6.61914 |
|  | Mature | 2.47902 | 0.34281 | 0.93082 | 7.58348 | 1.04223 | 5.37068 | 10.99694 | 10.37727 |
|  | Day 0-1 | 58.39756 | 0.00344 | 6.01623 | 0.74221 | 0.39960 | 0.04360 | 1.00000 | 0.23745 |
| **CTL** | Day 0-3 | 4.39572 | 0.07329 | 8.16674 | 0.48865 | 0.80491 | 1.00000 | 7.14410 | 0.05902 |
|  | Day 0-7 | 69.91616 | 1.01773 | 8.02566 | 1.00000 | 0.24257 | 1.07266 | 13.94112 | 6.61914 |
|  | Mature | 0.06305 | 0.00148 | 0.59201 | 0.45075 | 0.45403 | 1.54064 | 3.48770 | 0.31544 |
|  | Day 0-1 | 15.23940 | 0.03975 | 4.22119 | 3.18231 | 0.12948 | 0.21605 | 1.00000 | 1.00523 |
| **KTI** | Day 0-3 | 0.43044 | 0.21701 | 7.73616 | 2.70871 | 0.33754 | 1.00000 | 8.01503 | 0.15274 |
|  | Day 0-7 | 4.30063 | 1.01773 | 8.02566 | 1.00000 | 0.47333 | 0.86799 | 26.43356 | 6.61914 |
|  | Mature | 2.47902 | 0.00806 | 0.28050 | 1.60908 | 0.60088 | 2.57193 | 6.17954 | 0.78988 |
|  | Day 0-1 | 0.04075 | 0.28933 | 0.79471 | 10.10011 | 0.07243 | 0.18851 | 1.00000 | 1.21449 |
| **PDI** | Day 0-3 | 0.19218 | 0.32223 | 1.14564 | 15.03968 | 0.16723 | 1.00000 | 0.14641 | 1.00000 |
|  | Day 0-7 | 0.03079 | 1.01773 | 0.54481 | 1.00000 | 0.35274 | 0.95157 | 0.22858 | 1.43951 |
|  | Mature | 2.47902 | 0.12898 | 1.00000 | 3.52130 | 0.21968 | 0.17368 | 0.34077 | 3.97144 |
|  | Day 0-1 | 7.93868 | 0.00844 | 3.77334 | 14.55156 | 0.13566 | 0.01527 | 1.00000 | 1.12790 |
| **QKW** | Day 0-3 | 0.77522 | 0.14306 | 9.36326 | 4.67171 | 0.98082 | 1.00000 | 1.43131 | 0.10011 |
|  | Day 0-7 | 6.01302 | 1.01773 | 8.02566 | 1.00000 | 0.08780 | 1.29646 | 3.21928 | 6.61914 |
|  | Mature | 2.47902 | 0.00150 | 0.25606 | 12.00330 | 0.50227 | 2.48840 | 1.66478 | 0.87879 |
